# Tenofovir induced Fanconi syndrome in a middle age African female from Kenya, East Africa: Case report and brief literature review

**DOI:** 10.1002/ccr3.8889

**Published:** 2024-05-24

**Authors:** Fredrick Odhiambo, Shallot Nareeba, Grace Mwangeka, Ann Njambi, Vashti Nyakebati

**Affiliations:** ^1^ Maua Methodist Hospital Meru Kenya

**Keywords:** antiretroviral therapy, drug induced nephrotoxicity, Fanconi syndrome, tenofovir

## Abstract

This case presentation highlights the need to routinely monitor renal function in patients on Tenofovir Disoproxil Fumarate (TDF) due to its side effect of proximal tubule dysfunction.

**Abstract:**

This is a case presentation of a 50‐year‐old African female who had been on a Tenofovir based regimen for 12 years and developed Fanconi syndrome. She recovered after discontinuation of the Tenofovir Disoproxil Fumarate (TDF).

## 1 INTRODUCTION

Tenofovir Disoproxil Fumarate (TDF) is one of the newest and more tolerable backbone of highly active antiretroviral therapy (HAART) in the class of nucleotide reverse transcriptase inhibitor (NRTI). In 2016, World Health Organization (WHO) published a consolidated guideline that recommended Tenofovir based regimen as the first line for adults and adolescents and henceforward this regimen has been extensively rolled out.^1^ Despite its excellent safety profile and tolerability TDF is known to cause proximal tubule renal dysfunction. TDF renal tubulopathy can manifest as Fanconi syndrome (FS), Acute kidney injury or chronic kidney disease.^2^ Here, we present the case of a middle age African female on Tenofovir based regimen who developed FS after 12 years of Tenofovir based HAART.

## 2 CASE PRESENTATION

We present a case of a 50‐year‐old black African female on management of HIV for the last 12 years who presented to our hospital with complaints of longstanding generalized body aches and bone pains for a duration of 2 years. These symptoms got worse 3 weeks prior to admission and were associated with muscle weakness of both the upper and lower limbs. In addition, she had joint pains worsened by activity but not associated with stiffness. A year prior to admission she suffered a trivial fall without a fracture that rendered her unable to walk. She denied any of history of cough, weight loss, night sweats, back pain, or paraesthesia. Moreover, she had normal bladder and bowel control without polydipsia, polyuria or even polyphagia.

Our patient was initiated on HAART in 2011 and has been on her drugs with excellent adherence since then. She was on TDF/3TC/EFV, however in 2019 she was transferred to TDF/3TC/DTG as part of a Nationwide optimization program.

She has a history of pulmonary TB treatment 13 years ago but no history of any other opportunistic infection, hypertension or diabetes.

General exams revealed a middle‐aged female who was alert and responsive, groaning and moaning in pain. She was wasted with a BMI of 17.8 kg/m^2^ and a weight of 48 kg. There was no pallor, jaundice, dehydration, oedema, or lymphadenopathy. Her vital signs were within the normal ranges. On musculoskeletal exams she had reduced muscle bulk, reduced muscle power graded at 3/5 and tenderness on palpation of the muscles and along the long bones. Range of motion of the right hip joint was also restricted. The other systemic exams were normal.

## 3 METHODS

Laboratory investigations done at admission revealed reduced GFR at 35.80 mL/min/1.73m^2^ (CKD‐EPI) with raised creatinine levels of 152.58 umol/L. Her Calcium and Uric acid levels were low at 1.8 mmol/L and 2.26 mg/dL respectively. She had normal HBA_1c_ of 4.6% with a normal random blood sugar of 6.7 mmol/L and abnormal urinalysis findings that included glycosuria of 250 mg/dL, proteinuria of 100 mg/dL and urine pH of 7.0. She had normal full hemogram and electrolytes, and a negative Rheumatoid factor. Serum Protein Electrophoresis (SPEP) and Urine Protein Electrophoresis (UPEP) was normal with no paraproteinemia.

**FIGURE 1:**
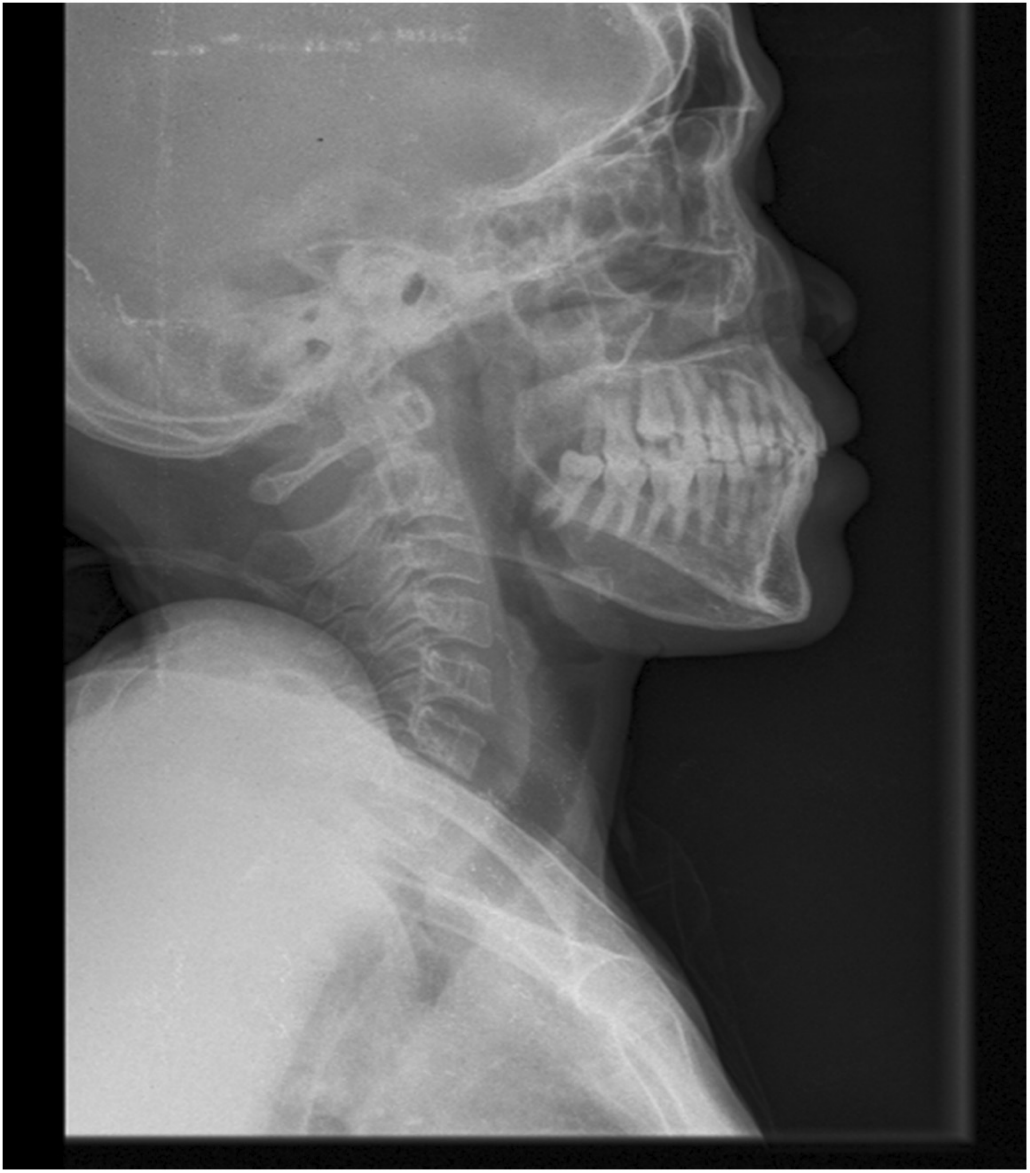
Skull radiograph reveals areas of radiolucency.

**FIGURE 2:**
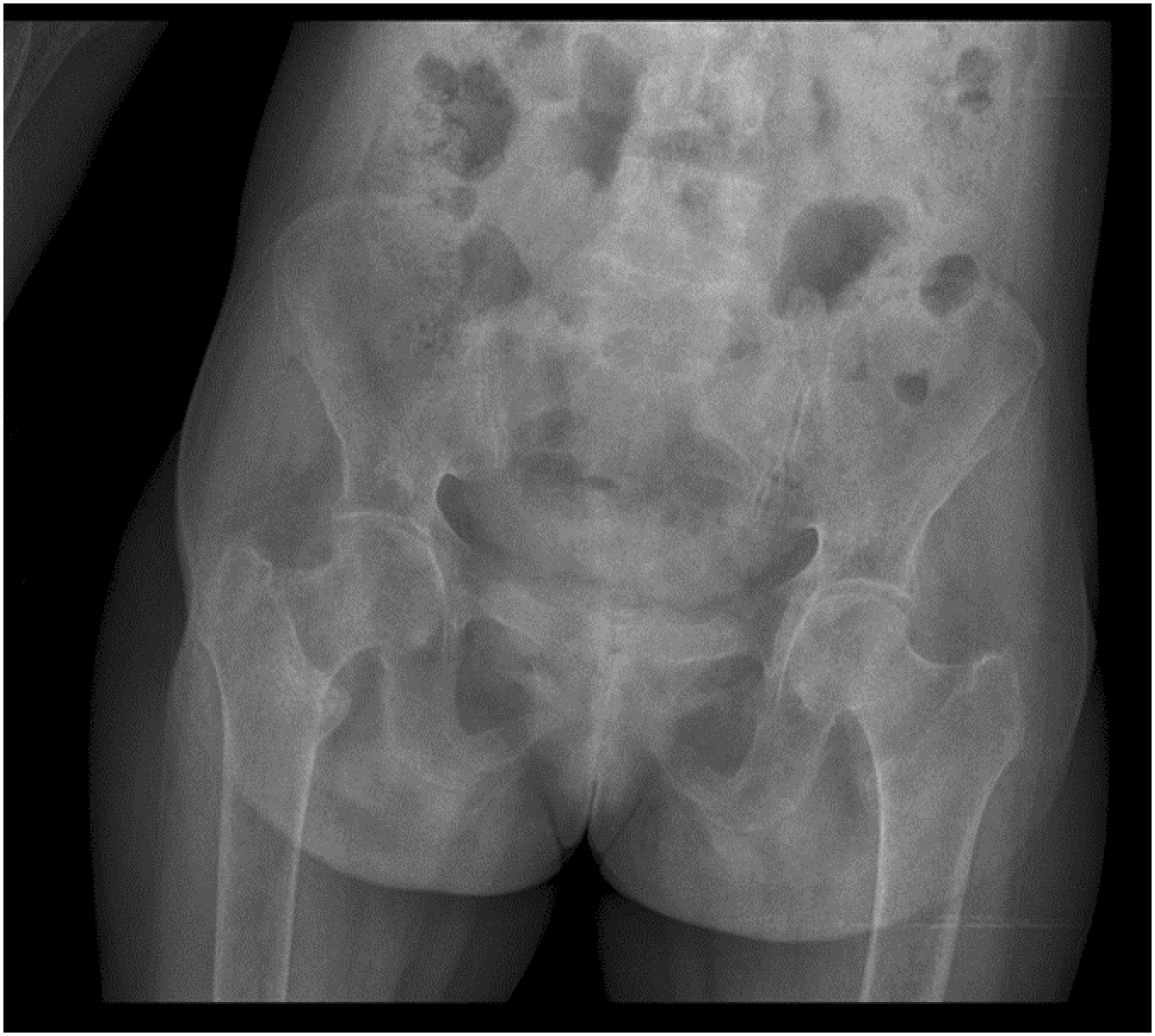
Pelvic radiograph reveals thinning of the cortical bone and loss of trabeculae especially at the proximal aspect of the femur.

KUB ultrasound was normal, but the bone survey x‐rays as depicted on Figure 1 and Figure 2, revealed features of osteoporosis. Pelvic Xray disclosed thinning of the cortical bone and loss of trabeculae especially at the proximal aspect of femur and skull Xray showed areas of radiolucency, all in keeping with osteoporosis. Renal biopsy was considered but due to financial constraints was not done.

A diagnosis of FS secondary to Tenofovir nephrotoxicity was made based on clinical symptoms of generalized body aches and bone pains and supporting laboratory findings of elevated creatinine levels, proteinuria, glycosuria, hypouricemia, osteoporosis, and a urine pH of >5.

Due to excellent adherence with undetectable viral load and cost implications on the patient, we stopped doing regular CD4 counts. CD4 cell count trends is depicted below on Table 1.

**TABLE 1:** CD4 cell counts trend.

Year	2010	2011	2012	2013	2014
CD4 count	136	523	323	340	400

## 4 CONCLUSION AND RESULTS

Our patient was then switched from TDF based regimen to an Abacavir based one, Abacavir/Lamivudine/ Dolutegravir. Serial creatinine levels were done, See Table 2.

**TABLE 2:** Creatinine trend.

Day	Admission	Day 10	Day 25	Day 81
Creatinine levels	152.58 umol/L	126.2 umol/L	118.7 umol/L	110 umol/L

She had progressive reduction in the creatinine levels and repeat of urinalysis 11 weeks later revealed clearance of the proteinuria and glycosuria, normal calcium levels of 2.25 mmol/L and improvement in muscle power and activities of daily living. She was now able to walk with support using a walker.

## 5 DISCUSSION

TDF is largely renally excreted via active tubular secretion and glomerular filtration in its unchanged form with no interaction with the CYP450 pathway.^3^ 20%–30% of the drug is excreted via tubular secretion.^4^ The main mechanism of proximal tubular injury results from mitochondrial destruction following an imbalance between intracellular uptake and efflux of tenofovir^5^ mediated by human organic anion transporters (hOAT) and multidrug resistance proteins (MRPs) respectively that leads to intracellular accumulation of TDF. A decreased glomerular filtration rate (GFR) has been shown to be associated with increased intracellular concentrations of TDF, through increased OAT1 activity. With intracellular toxicity, structural mitochondrial damage results from loss of mitochondrial proteins and DNA. The resultant mitochondrial depletion and dysfunction triggers apoptosis of the cell.^6^

FS is a proximal tubule dysfunction that results in normoglycemic glycosuria, as well as urinary loss of phosphate, calcium, uric acid, amino acids, bicarbonates, and tubular proteins.^7^ Tenofovir can cause complete or partial FS. Majority of cases present as partial with an elevation in creatinine levels, hypophosphatemia, and glycosuria.^8^ Patients on TDF based regimen are at five times greater risk of developing chronic kidney disease in comparison to patients on a non‐TDF based regimen. This rapid decline of estimated GFR mostly occur in the first 2–3 years of treatment.^9^ The prevalence of renal dysfunction induced by TDF is estimated at 5.6%, and the risk is increased with advanced age, low BMI, low baseline CD4 count, hypertension, and diabetes.^10^ Severe nephrotoxicity to warrant discontinuation has been reported in 1% patients yearly.^11^ Africans with pre‐existing renal disease and advanced age are at a greater risk of statistically significant TDF associated renal function decline.^12^ Concomitant protease inhibitor use has also been suggested to contribute to development of TDF associated nephrotoxicity.^13^ Other factors including low body weight and low CD4 cell count have been linked to increased susceptibility of TDF tubulopathy in some individuals.^14^ The diagnostic criteria for TDF related FS include normoglycemic glycosuria, proteinuria, and hypophosphatemia with phosphaturia^15^ that can be screened for, through urine and blood tests. Reliance on elevations in eGFR and urine albumin/creatinine ratio, may lead to missed diagnoses due to their poor sensitivity as markers of proximal tubular function.^16^

Approximately half of the patients attain partial or full recovery of renal function after 1 year of TDF discontinuation, defined as >70% of pre‐TDF creatinine clearance with majority of major markers of proximal tubulopathy resolving within 8 weeks of drug discontinuation.^17^ However, full reversibility of TDF –related renal toxicity is not always the rule.^18^ Early switching of TDF in patients with proximal renal tubulopathy has been associated with better chances of complete renal recovery as well as low levels of urine dipstick proteinuria at the time of discontinuation.^19^

This case illustrates a patient from an underserved region of a Low Middle‐Income Country on Tenofovir based regimen for 12 years. She has excellent adherence as confirmed by the consistent undetectable viral load. However, she is unable to afford annual renal function tests as recommended by HAART guidelines.^8^ Renal function should be monitored prior to starting and throughout therapy as clinically appropriate, and caution should be taken with administering TDF with combination with other potentially nephrotoxic agents.^20^ HIV Medicine Association of the IDSA recommend at least biannual monitoring of renal function, serum phosphorus, proteinuria, and glycosuria in patients receiving tenofovir with GFR <90 mL/min/1.73 m^2^, other comorbid diseases or cotreated with protease inhibitors, due to the risk of nephrotoxicity.^13^

It follows a trivial fall with persistent and worsening body and bone pains that the patient gets to be admitted. Further work up revealed normoglycemic glycosuria, increase in creatinine with declining GFR, proteinuria, hypouricemia, hypocalcemia, and osteopenia. These laboratory findings are in keeping with FS where 5 out of 7 nondiabetic patients biopsied for Tenofovir nephrotoxicity had glycosuria with increased serum creatinine.^21^ Inability to do Arterial Blood Gases (ABG) and phosphates in the resource limited regions further stifles the ability to clinch the diagnosis earlier.^22^ The bone pain and generalized body aches are possibly a consequence of osteomalacia, which is a late manifestation of proximal tubulopathy secondary to phosphate wasting and/calcitriol deficiency since calcitriol is synthesized by the mitochondria in the proximal tubules.^23^ About 40%–90% of HIV infected individuals experience low bone mineral density consistent with the diagnosis of osteopenia or osteoporosis.^24^ The cause of this multifactorial but antiretroviral toxicity, particularly TDF, has been implicated.^25^ Potential mechanisms of TDF toxicity on the bone can be due to direct or indirect effect. In vitro studies have demonstrated altered expression of genes involved in cell signaling, energy and amino acid metabolism in osteoclasts and osteoblasts exposed to physiological doses of TDF, a direct negative effect.^26^ Indirect effects are via renal/endocrine systems, severe tubulopathy due to TDF can result in bicarbonate and phosphate wasting that leads to osteomalacia and bone pain.^27^

TDF may also affect vitamin metabolism directly driving a state of sustained hyperparathyroidism and increased bone turnover, in subjects on stable TDF containing ART, higher plasma TDF level have been associated with higher levels of vitamin D binding receptor leading to lower free 1, 25‐hydroxy‐vitamin D.^28^ This “functional” deficiency can drive secondary hyperparathyroidism in patients on TDF containing regimens. Due to this there is therapeutic role for vitamin D3 supplementation at initiation of TDF containing ART to attenuate increase in PTH and reduction in bone mineral density.^29^

The proposed risk factors in this case includes and is not limited to prolonged TDF use,^30^ low body weight and advanced age.^14^ The patient symptoms and renal function markedly improved following TDF discontinuation. 6 weeks later, she had no glycosuria, proteinuria, hypocalcemia and had reduced creatinine levels. Alexandre Karras et al^31^ reported that most of laboratory values in TDF tubulopathy returned to normal following its discontinuation.

In conclusion, we would like to emphasize the need for routine monitoring of renal function of patients on TDF based regimen for nephrotoxicity, even years after initiation of drugs. On any occasion TDF tubulopathy signs are recognized, the drug should be stopped to prevent further complications.

## AUTHOR CONTRIBUTIONS

**Fredrick Odhiambo:** Conceptualization; formal analysis; investigation; methodology; resources; supervision; writing – original draft; writing – review and editing. **Shallot Nareeba:** Conceptualization; investigation; methodology; writing – original draft; writing – review and editing. **Grace Mwangeka:** Conceptualization; investigation; resources; writing – original draft; writing – review and editing. **Ann Njambi:** Conceptualization; investigation; supervision; writing – review and editing. **Vashti Nyakebati:** Supervision; writing – original draft; writing – review and editing.

## CONFLICT OF INTEREST STATEMENT

The authors of this case report declare that it was conducted in absence of any commercial or financial relationships that could be construed as a potential conflict of interest.

## CONSENT

Written informed consent was obtained from the patient to publish this report in accordance with the journal’s patient consent policy.

## Data Availability

The data that support the findings of this case report are available from the corresponding lead author upon reasonable request. The data are not publicly available due to patient's privacy and ethical restrictions. The case report includes all relevant anonymized patient data and clinical findings related to the diagnosis and management of tenofovir induced Fanconi syndrome. Further inquiries can be directed to the corresponding lead author, Fredrick Otieno.

## References

[1] WHO . Updated recommendations on the first line and second line antiretroviral regimens and post exposure prophylaxis and recommendations on early infant diagnosis of HIV. 2018.

[2] Verhelst D , Monge M , Meynard JL , et al. Fanconi syndrome and renal failure induced by tenofovir: a first case report. Am J Kidney Dis. 2002;40:13313. 10.1053/ajkd.2002.36924 12460055

[3] Venter W , Fabian J , Feldman C . An overview of tenofovir and renal disease for the HIV‐treating clinician. Southern African J HIV Med. 2018;19(1):8. doi:10.4102/sajhivmed.v19i1.817 PMC6111387 30167339

[4] Kwiatkowska E , Domański L , Dziedziejko V , Kajdy A , Stefańska K , Kwiatkowski S . The mechanism of drug nephrotoxicity and the methods for preventing kidney damage. Int J Mol Sci. 2021;22(11):6109. doi:10.3390/ijms22116109 34204029 PMC8201165

[5] Wassner C , Bradley N , Lee Y . A review and clinical understanding of tenofovir: tenofovir disoproxil fumarate versus tenofovir alafenamide. journal of the international association of providers of. AIDS Care. 2020;19: doi:10.1177/2325958220919231 PMC7163232 32295453

[6] Jafari A , Khalili H , Dashti‐Khavidaki S . Tenofovir‐induced nephrotoxicity: incidence, mechanism, risk factors, prognosis and proposed agents for prevention. Eur J Clin Pharmacol. 2014;70(9):1029‐1040. doi:10.1007/s00228-014-1712-z 24958564

[7] Izzedine H , Launay‐Vacher V , Isnard‐Bagnis C , Deray G . Drug‐induced Fanconi's syndrome. Am J Kidney Dis. 2003;41:(pg. 292–309). 10.1053/ajkd.2003.50037 12552490

[8] Izzedine H , Hulot JS , Vittecoq D , et al. Long‐term renal safety of tenofovir disoproxil fumarate in antiretroviral‐naive HIV‐1‐infected patients: data from a double‐blind randomized active‐controlled multicentre study. Nephrol Dial Transplant. 2005;20:743. 15741212 10.1093/ndt/gfh658

[9] Jirayu Visuthranukul TR . Incidence rate and time to occurrence of renal impairment and chronic kidney disease among thai hiv‐infected adults with tenofovir disoproxil fumarate use. Open Aids J. 2021;15:73‐80.

[10] Nagalingeswaran Kumarasamy SS . Prevalence and factors associated with renal dysfunction in patients on tenofovir disoproxil fumarate‐based antiretroviral regimens for HIV infection in Southern India. J Virus Erad. 2018;4(1):37‐40. Retrieved from. doi:10.1016/S2055-6640(20)30245-4 29568552 PMC5851183

[11] Fux CA , Simcock M , Wolbers M , et al. Tenofovir use is associated with a reduction in calculated glomerular filtration rates in the Swiss HIV Cohort Study. Antivir Ther. 2007;12(8):1165‐1173. 18240857

[12] Mtisi TJ , Ndhlovu CE , Maponga CC , Morse GD . Tenofovir‐associated kidney disease in Africans: a systematic review. AIDS Res Ther. 2019;16:12. doi:10.1186/s12981-019-0227-1 31171021 PMC6554992

[13] Ryan D . Systematic review and meta‐analysis: renal safety of tenofovir disoproxil fumarate in hiv‐infected patients. Clin Infect Dis. 2010;51(5):496‐505. doi:10.1086/655681 20673002

[14] Nelson MR , Katlama C , Montaner JS , et al. The safety of tenofovir disoproxil fumarate for the treatment of HIV infection in adults: the first 4 years. AIDS (London, England). 2007;21(10):1273‐1281. doi:10.1097/QAD.0b013e3280b07b33 17545703

[15] Rao M , Dadey L , Glowa T , Veldkamp P . Fanconi Syndrome Leading to Hypophosphatemic Osteomalacia Related to Tenofovir Use. Infectious disease reports. 2021;13(2):448‐453. doi:10.3390/idr13020044 34073672 PMC8162330

[16] Mothobi NZ , Masters J , Marriott DJ . Fanconi syndrome due to tenofovir disoproxil fumarate reversed by switching to tenofovir alafenamide fumarate in an HIV‐infected patient. Thera Adv Infec Dis. 2018;5(5):91‐95. doi:10.1177/2049936118785497 PMC6136117 30224952

[17] Gupta SK , Anderson AM , Ebrahimi R , et al. Fanconi syndrome accompanied by renal function decline with tenofovir disoproxil fumarate: a prospective, case‐control study of predictors and resolution in HIV‐infected patients. PLoS One. 2014;9(3):1‐7. Retrieved from. 10.1371/journal.pone.0092717 PMC3961428 24651857

[18] Wever K , van Agtmael MA , Carr A . Incomplete reversibility of tenofovir‐related renal toxicity in HIV‐infected men. J Acquir Immune Defic Syndr. 2010;55(1):78‐81. 10.1097/QAI.0b013e3181d05579 20173649

[19] Patamatamkul S , Songumpai N , Payoong P , Katavetin P , Putcharoen O . Early switching of tenofovir disoproxil fumarate (TDF) in HIV‐infected patients with TDF‐induced nephrotoxicity: a prospective study. HIV research & clinical practice. 2022;23(1):99‐106. 36065999

[20] Fernandez BF , Ferrer AM , Sanz AB , et al. Tenofovir nephrotoxicity. AIDS Res Treat. 2011;2011:1‐11. 10.1155/2011/354908 PMC3119412 21716719

[21] Herlitz LC , Mohan S , Stokes MB , Radhakrishnan J , D'Agati VD , Markowitz GS . Tenofovir nephrotoxicity: acute tubular necrosis with distinctive clinical, pathological, and mitochondrial abnormalities. Kidney Int. 2010;78(11):1171‐1177. 20811330 10.1038/ki.2010.318

[22] Badiou S , Merle De Boever C , Terrier N , Baillat V , Cristol JP , Reynes J . Is tenofovir involved in hypophosphatemia and decrease of tubular phosphate reabsorption in HIV‐positive adults? J Infect. 2006;52(5):335‐338. 16176835 10.1016/j.jinf.2005.07.020

[23] Perrot S , Aslangul E , Szwebel T , Caillat‐Vigneron N , Le Jeunne C . Bone pain due to fractures revealing osteomalacia related to tenofovir‐induced proximal renal tubular dysfunction in a human immunodeficiency virus‐infected patient. J Clin Rheumatol. 2009;15(2):72‐74. 19265350 10.1097/RHU.0b013e31819c20d8

[24] Brown TT , McComsey GA , King MS , Qaqish RB , Bernstein BM , da Silva BA . Loss of bone mineral density after antiretroviral therapy initiation, independent of antiretroviral regimen. J Acquir Immune Defic Syndr. 2009;51(5):554‐561. [PubMed] [Google Scholar]. 19512937 10.1097/QAI.0b013e3181adce44

[25] Grant PM , Cotter AG . Tenofovir and bone health. Curr Opin HIV AIDS. 2016;11(3):326‐332. doi:10.1097/COH.0000000000000248 26859637 PMC4844450

[26] Grigsby IF , Pham L , Mansky LM , Gopalakrishnan R , Carlson AE , Mansky KC . Tenofovir treatment of primary osteoblasts alters gene expression profiles: implications for bone mineral density loss. Biochem Biophys Res Commun. 2010;394(1):48‐53. [PMC free article] [PubMed] [Google Scholar] [Ref list]. 20171173 10.1016/j.bbrc.2010.02.080 PMC2847063

[27] Rodriguez‐Novoa S , Labarga P , Soriano V , et al. Predictors of kidney tubular dysfunction in HIV‐infected patients treated with tenofovir: a pharmacogenetic study. Clin Infect Dis. 2009;48(11):e108‐e116. [PubMed] [Google Scholar] [Ref list]. 19400747 10.1086/598507

[28] Havens PL , Kiser JJ , Stephensen CB , et al. Association of higher plasma vitamin D binding protein and lower free calcitriol levels with tenofovir disoproxil fumarate use and plasma and intracellular tenofovir pharmacokinetics: cause of a functional vitamin D deficiency? Antimicrob Agents Chemother. 2013;57(11):5619‐5628. [PMC free article] [PubMed] [Google Scholar] [Ref list]. 24002093 10.1128/AAC.01096-13 PMC3811269

[29] Havens PL , Stephensen CB , Hazra R , et al. Vitamin D3 decreases parathyroid hormone in HIV‐infected youth being treated with tenofovir: a randomized, placebo‐controlled trial. Clin Infect Dis. 2012;54(7):1013‐1025. [PMC free article] [PubMed] [Google Scholar] [Ref list]. 22267714 10.1093/cid/cir968 PMC3297650

[30] Woodward C , Hall A , Williams I . Tenofovir‐associated renal and bone toxicity. HIV Med. 2009;10(8):482‐487. 19459988 10.1111/j.1468-1293.2009.00716.x

[31] Karras A , Lafaurie M , Furco A , et al. Tenofovir‐related nephrotoxicity in human immunodeficiency virus‐infected patients: three cases of renal failure, Fanconi syndrome, and nephrogenic diabetes insipidus. Clin Infect Dis. 2003;36(8):1070‐1073. doi:10.1086/368314 12684922

